# Perturbation of Thymocyte Development Underlies the PRRS Pandemic: A Testable Hypothesis

**DOI:** 10.3389/fimmu.2019.01077

**Published:** 2019-05-15

**Authors:** John E. Butler, Marek Sinkora, Gang Wang, Katerina Stepanova, Yuming Li, Xuehui Cai

**Affiliations:** ^1^Carver College of Medicine, University of Iowa, Iowa, IA, United States; ^2^Laboratory of Gnotobiology, Institute of Microbiology of the Czech Academy of Sciences, Prague, Czechia; ^3^State Key Laboratory of Veterinary Biotechnology, Harbin Veterinary Research Institute, Chinese Academy of Agricultural Sciences, Harbin, China

**Keywords:** hypergammaglobulinemia, PRRS virus, T cell repertoire, thymic atrophy, hypothesis

## Abstract

Porcine reproductive and respiratory syndrome virus (PRRSV) causes immune dysregulation during the Critical Window of Immunological Development. We hypothesize that thymocyte development is altered by infected thymic antigen presenting cells (TAPCs) in the fetal/neonatal thymus that interact with double-positive thymocytes causing an acute deficiency of T cells that produces “holes” in the T cell repertoire allowing for poor recognition of PRRSV and other neonatal pathogens. The deficiency may be the result of random elimination of PRRSV-specific T cells or the generation of T cells that accept PRRSV epitopes as self-antigens. Loss of helper T cells for virus neutralizing (VN) epitopes can result in the failure of selection for B cells in lymph node germinal centers capable of producing high affinity VN antibodies. Generation of cytotoxic and regulatory T cells may also be impaired. Similar to infections with LDV, LCMV, MCMV, HIV-1 and trypanosomes, the host responds to the deficiency of pathogen-specific T cells and perhaps regulatory T cells, by “last ditch” polyclonal B cell activation. In colostrum-deprived PRRSV-infected isolator piglets, this results in hypergammaglobulinemia, which we believe to be a “red herring” that detracts attention from the thymic atrophy story, but leads to our second independent hypothesis. Since hypergammaglobulinemia has not been reported in PRRSV-infected conventionally-reared piglets, we hypothesize that this is due to the down-regulatory effect of passive maternal IgG and cytokines in porcine colostrum, especially TGFβ which stimulates development of regulatory T cells (Tregs).

## Background and Hypothesis

Porcine reproductive and respiratory syndrome (PRRS) is a major threat to swine health and global pork production. It is considered responsible for an annual 660 million dollar loss to the pork industry in the USA alone with proportionally similar loses in other countries (1; Lager this volume). The disease is a pandemic and ~25 years of research has yet to clearly define the immune pathology that allows the virus to persist in young pigs for up to 150 dpi (1). Therefore, we believe it is time to offer a testable hypothesis to explain the immune pathogenesis and persistence of PRRS in the belief that combating the PRRS pandemic and engineering vaccines depend on identifying the cause of the immune dysregulation. We believe that attenuated viral vaccines will have limited success unless they prevent infection of thymic antigen presenting cells (TAPCs) during the period in which the T cell repertoire is being developed.

PRRS is caused by a member of the Arteriviridae, order Nidoviralies, which includes lactate dehydrogenase elevating virus in mice (LDV), equine arterivirus (EAV), and simian hemorrhagic fever disease (SHFV). The virus is trophic for macrophages and dendritic cells, whereon, CD163 serve as a receptor (2) and when deleted, prevents macrophages infection (3). As indicated by the name, Porcine reproductive and respiratory syndrome virus (PRRSV) causes both fetal abortion and respiratory disease. Neonates are especially susceptible to viral and bacterial pathogens, because they encounter them during a critical period in development. The situation with PRRS is made more difficult in Class III Artiodactyls like swine (4, 5) because the virus can cross the placenta but protective maternal antibodies or cytotoxic T cells (CTLs) cannot. The mechanism of placental transfer of the virus is unclear, but may involve infected macrophages as is the case with LDV (6). Transfer may be facilitated by virus-induced apoptosis at the maternal-fetal interface (7). In any case, the primary target in fetuses is the thymus (8). Not surprisingly, fetal piglets develop the same features of immune dysregulation as seen in isolator piglets (9, 10). In contrast to piglets, infected adult swine make effective VN antibodies and can eliminate the infection (11) and VN antibodies from convalescent sows experimentally administered to piglets provide sterilizing immunity (12). These observation indicate that PRRS is a fetal/ newborn disease that strikes during the Critical Window of Immunological Development (13).

Early reports on PRRS showed that PRRSV-infected fetal and newborn piglets had increased susceptibility to secondary pathogens (14–17). More recent observations support this view (18–21). Co-infection studies using swine influenza (SIV), porcine circovirus Type 2 (PCV-2), *Salmonella choleraesuis, Mycoplasma hyopneumoniae*, and *Streptococcus suis* all result in prolonged fever and respiratory distress in PRRSV-infected piglets compared to controls infected with these pathogens alone. Twenty of 22 PRRS piglets co-infected with *S. suis* died, but only 5 of 23 infected with *S. suis* (22). Anti-PRRSV antibodies can be detected 6–14 dpi (22, 23) but VN antibodies do not appear before 28 dpi or later (24, 25), reminiscent of lymphocyte choriomeningitis virus (LCMV) infections in mice (26). Thus, the lack of VN antibodies when they are most needed, is one feature of this persistent viral disease. These observations collectively suggest that PRRSV infection induces immune suppression, i.e., neonatal immune dysregulation. This seems consistent with the acute lymphopenia after infection (27–30) although this can also occurs in many infectious diseases as monocytes and lymphocytes translocate from blood to hard tissue sites.

**Figure 1:**
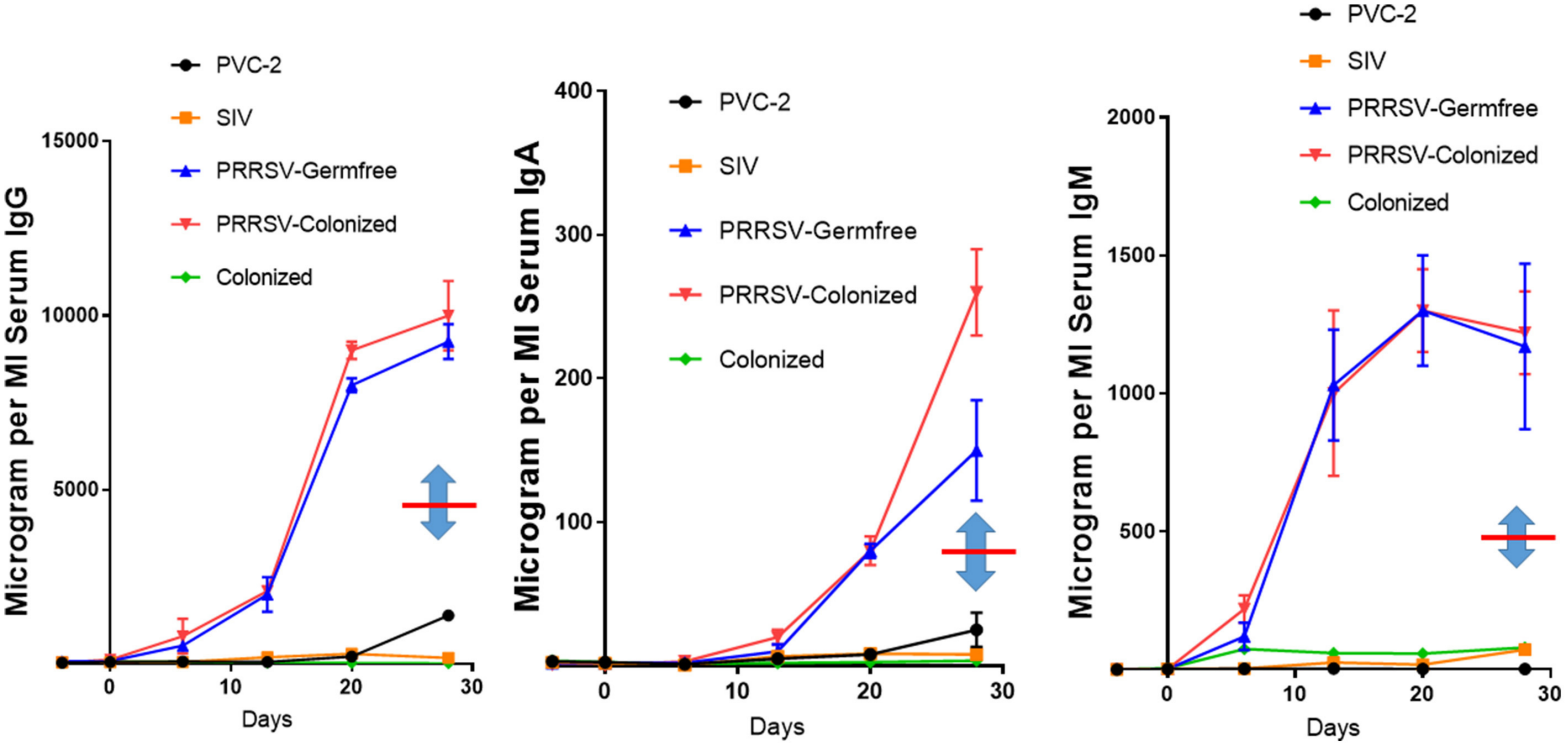
PRRSV-induced hypergammaglobulinemia in isolator piglets. Serum IgG, IgA, and IgM levels during 4 weeks post infection in isolator piglets infected with low pathogenic PRRSV strain VR-2332, PCV-2, SIV, and germfree controls. Also shown are levels in PRRSV-infected isolator piglets that were colonized with benign *E. coli*. Double arrows indicate the mean Ig levels at 28 days postpartum in conventionally-reared piglets. Error bars are SEM. Those for PCV-2 and SIV are often smaller than the symbol and were therefore omitted. Legend is on the figure.

Some features of immune dysregulation are exaggerated in piglets reared in isolator units that are denied access to maternal colostrum and a natural gut flora. These piglets develop severe hypergammaglobulinemia, exhibit lymph node hyperplasia, develop lung lesions while autoantibodies appear and immune complexes are deposited in their kidneys and vasculature (9). Figure 1 shows that IgG levels are elevated ~20-fold and IgA and IgM levels are elevated 10-fold in PRRSV-infected piglets vs. littermates infected with SIV and PCV-2. While data on serum Ig levels in conventionally-reared piglets is limited, there are no reports of hypergammaglobulinemia. In any case, comparison of conventionally-reared piglets with isolator piglets indicates that IgG, IgM, and IgA levels in serum during hypergammaglobulinemia are circa 2-fold higher than in conventionally-reared piglets (Figure 1, note double arrows). Colonization with benign *E. coli* does not reduce the degree of hypergammaglobulinemia (Figure 1) yet the same benign *E. coli* stimulates development of their adaptive immune system (31).

**Figure 2:**
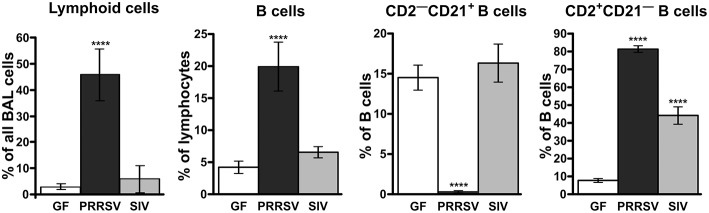
Analysis of lymphoid cells and B lymphocyte subsets from the bronchio-alveolar lavage following infections of isolator piglets with PRRSV VR-2332 strain and SIV. Cell suspensions were analyzed according to phenotype using flow cytometry. The CD2^−^CD21^+^ subpopulation is comprised of primed/activated while CD2^+^CD21^−^ are memory/plasma cells. The functional role of these subpopulations has been described (32, 33). Statistically significant difference from GF animals are indicated by asterisks.

Consistent with hypergammaglobulinemia in isolator piglets, PRRSV strongly stimulates B cell activity resulting in swift differentiation of naive CD2^+^CD21^+^ B cells to CD2^+^CD21^−^ antibody-forming cells (AFC) seemingly by-passing the presence of primed CD2^−^CD21^+^ B cells [Figure 2; (34)]. This decrease in CD21^+^ B cells has also been observed in PRRSV-infected conventional piglets (25, 28, 29). In isolator piglets this results in a 5-fold increase in circulating B cells and high levels of Ig producing cells in secondary lymphoid tissues compared to infection with SIV and PCV-2. This increase is especially notable 3 weeks after infection and is inversely correlated with a shift from CD4^+^CD8^−^ αβ T cells to double positive (DP) T cells (see later). We believe that rapid differentiation in the B cell compartment of PRRSV-infected isolator piglets explains the hypergammaglobulinemia of all isotypes (Figure 1). Such a rapid differentiation process would seem to leave little time for effective germinal center (GC) activity in lymph nodes that require virus-specific helper T cells to select B cells with high affinity BCRs for PRRSV epitopes. The polyclonal expansion of the major Vβ families suggests that the extraordinary B cell expansion must be is driven by non-specific helper T cells rather than the few that are virus-specific (34).

**Figure 3:**
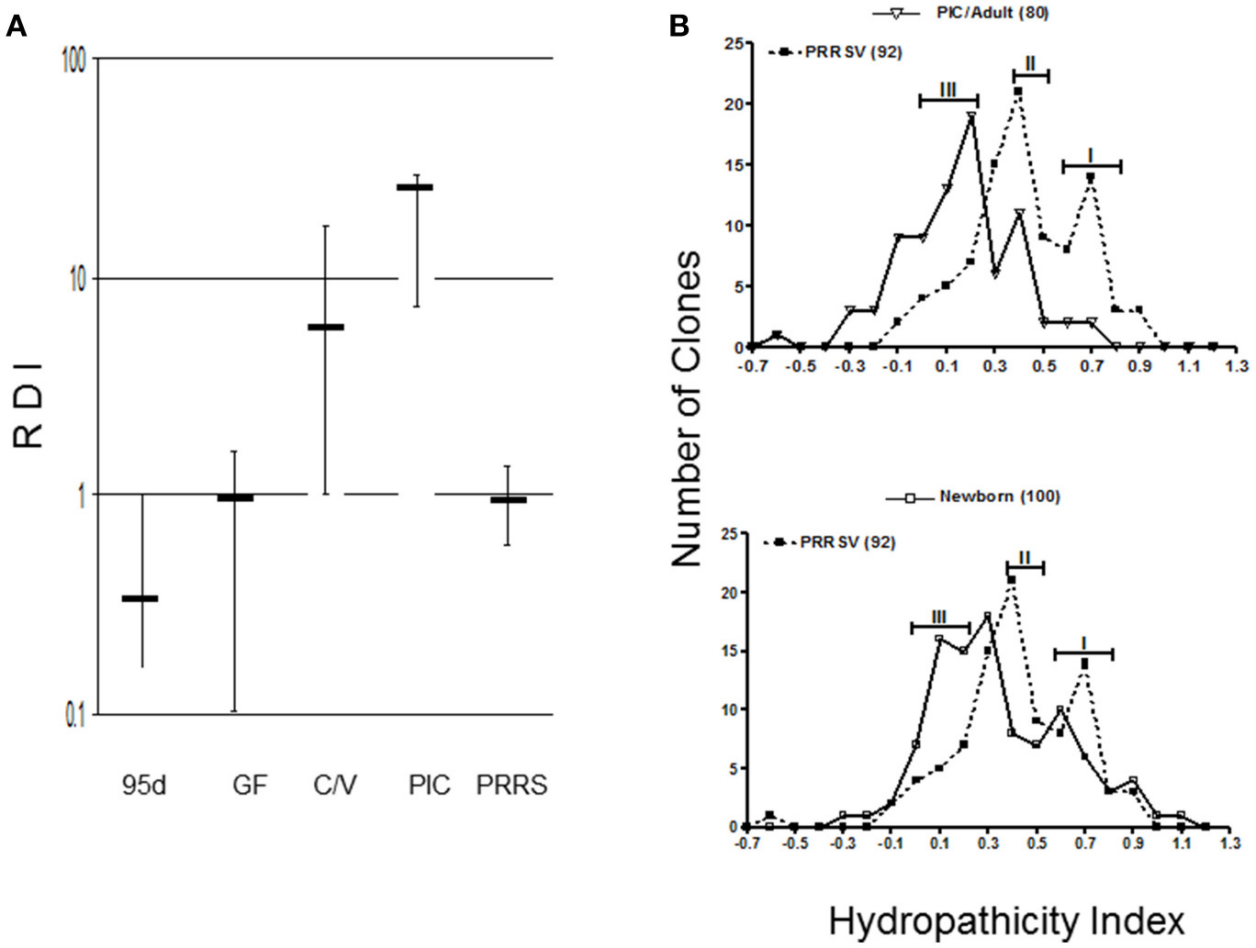
Antibody repertoire development in PRRSV-infected isolator piglets. **(A)** Repertoire development measured as a repertoire diversification index (RDI) for various piglet groups in which error bars represent the SEM. PIC, antigenized adults; C/V, colonized SIV-infected piglets; 95d, fetal piglets at 95 days of gestation. **(B)** Hydropathicity profiles for HVCDR3 of PRRSV strain VR-2332 -infected piglets compared to antigenized adults (PIC) or GF newborn piglets. Numbers in parentheses indicate the number of clones sequenced. [From Butler et al. (35)].

Although the number of lymph node GC has not been quantified or characterized in PRRS, the scenario we describe predicts that antibody repertoire development in PRRSV-infected piglet would be poor or retarded since their Repertoire Diversification Index (RDI) is indistinguishable from that of fetal and germfree piglets [(35); Figure 3A]. Thus, germline B cell populations that differentiate to AFC cells do so with limited selection for antigen specificity. This is supported by comparative spectratypic studies of the CDR3 region of heavy chain variable region genes (HVCDR3). These show that the HVCDR3 spectrum in PRRSV-infected piglets resembles the unselected spectrum of germfree piglets whereas that for SIV and PCV-2 infected piglets shows selection of specific clones (34). Analyses of VDJ sequences from PRRSV-infected piglets display the strongly hydrophobic characteristic of an undiversified antibody repertoire [(36, 37); Figure 3B]. An analysis of >415 HVCDR3 sequences showed that when examined in a hydropathicity profile, 92 sequences from PRRSV-infected piglet gave peaks at 0.4 and 0.7 which most resembled newborn piglets whereas in adults and SIV-infected controls, the index shifted to 0.1–0.3 with a loss of clones in the 0.5–0.9 range. In the case of PRRS, the hydrophobicity appears dependent on the use of RF3 of DHA (HVD1) and many transcripts display the AMVLV motif. Thus, antibodies with hydopathicities of 0.4 and 0.7 are products of B cells that like those in newborns, have not diversified their repertoire under pressure from antigen and antigen-specific helper T cells.

In a polar environment, antibodies with hydrophobic binding sites may result in aggregation and give rise to what some call hydrophobic immune complexes that bind to ELISA plates even in the presence of Tween 20 (38, 39). Whether these are indeed immune complexes or IgG aggregates has not been tested. In any case, they are also deposited in the vasculature and kidney (9). Authentic autoantibodies are also a feature of PRRS (9) and LDV in mice (40) and include antinuclear antibodies and those directed to the Golgi. Whether aggregates or true autoantibodies, both phenomena are signs of immune dysregulation. Further support for immune dysregulation is that IDEXX tests indicate that < 1% of IgG in PRRSV-infected isolator piglets is specific to PRRSV and are merely the consequence of non-specific B cell activity and may be of little virus-protective value (9).

**Figure 4:**
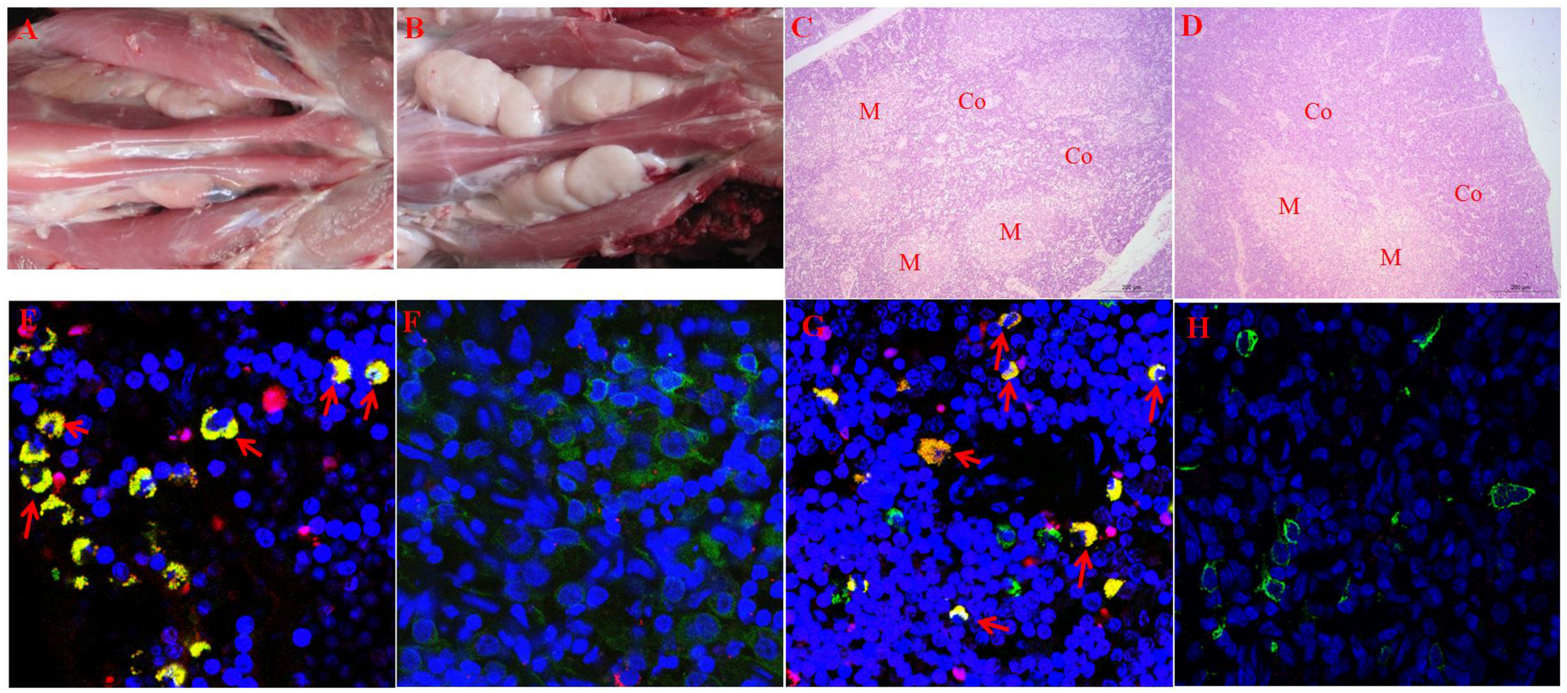
Thymic atrophy and localization of PRRSV in thymic APCs. **(A–D)** (upper tier). Macroscopic and histological evidence of thymus atrophy in which **(A,C)** are from infected piglets. Thymocytes in the cortex (Co) are depleted and replaced by granulocytes. M, medulla. **(E–H)** (Lower tier). Immunohistology of infected and control thymi. In all sections, blue, nuclei stained with DAPI. **(E,G)** are from piglets infected with the high path HuN4 strain while **(F,H)** are from control piglets. In **(E,F)**, apoptotic cells are red, CD3+ cells are green and double-strained cells (green + red) are yellow. In sections **(G,H)**, CD14+ APC are green, those containing the PRRSV N protein are red so that infected APCs are yellow. [From Wang et al. (43), He et al. (44), and Li et al. (45)].

**Figure 5:**
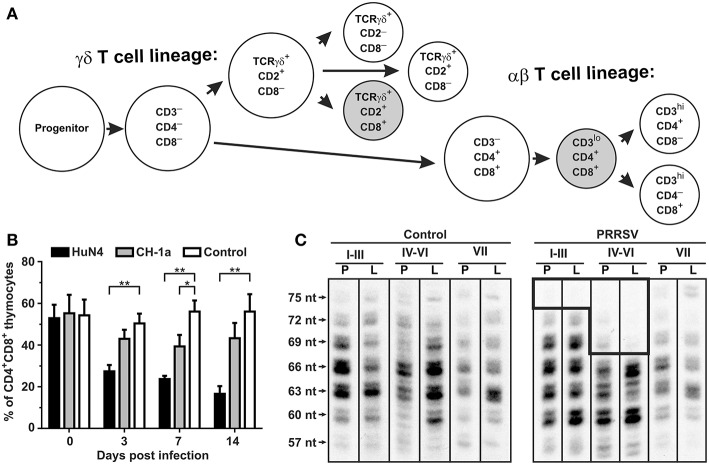
Consequence of PRRSV infection in the porcine thymus and periphery. **(A)** Differentiation pathways of αβ and γδ thymocytes in swine (47–51). Shaded subsets are those which are significantly decreased during infection with low pathogenic VR-2332. **(B)** Flow cytometric comparison of non-infected (control) and PRRSV infected pigs with high-pathogenic strain HuN4 and low-pathogenic strain CD-1a shows that CD4^+^CD8^+^ thymocytes of αβ lineage are depleted after PRRSV infection depending on their pathogenicity (44). Asterisks (^* and **^) denote significance at the 0.05 and 0.01 levels, respectively. **(C)** Analysis of peripheral αβ T cells by CDR3 length analysis (52) of T cell repertoire (TCRBV) isolated from peripheral blood (indicated by P) and Broncho alveolar lavage (indicated by L) from piglets infected with the VR-2332 strain is also shown. Analysis done for VβI–VβIII families (I–III), VβIV–VβVI families (IV–VI), and VβVII family (VII) (47). The hole in TCRβ repertoire in PRRSV infected animals is boxed. Lengths of CDR3 are indicated on left and include number of nucleotides (nt) from the 3′end of V segment to the 3′end of J segment (34).

The hypergammaglobulinemia seen in PRRSV-infected isolator piglets was a striking distraction in swine immunology until it was realized that especially polyclonal B cell activation and sometimes hypergammaglobulinemia, are common in numerous viral, bacterial and parasitic infections (41). While lymphopenia, lack of VN antibodies and hypergammaglobulinemia are symptoms of immune dysregulation, these alone are unlikely to be the cause of viral persistence. Rather there is a more compelling feature of PRRSV pathology which dates to the earliest observations on PRRSV-induced pathology that identified the thymus as one of the target organs (8, 22) and in extreme cases, reported its complete absence (41, 42). This correlated with a delayed antibody response and a decrease in helper cell-associated IL-4 (42). Thymus atrophy (Figures 4A,C) can explain PRRS-related lymphopenia (27, 28, 30, 42) and its severity is directly correlated with strain virulence (44, 46). Immunohistochemical studies indicate that thymocytes are undergoing apoptosis (Figure 4F) and flow cytometric studies show that DP thymocytes are depleted (Figures 5A,B). Depletion of DP thymocytes is greatest with the highly virulent HuN4 strain (Figure 5B) which explains why the relative proportion of immature CD4^+^ and CD8^+^ cells in the thymus is elevated (44). Histological studies show that the PRRSV nuclear antigen is localized to CD14^+^ thymic cells [Figure 4G; (45)]. The latter observation is not surprising since PRRSV infects monocyte lineage cells and there is no evidence that cells other than TAPCs are involved in thymocyte development (53, 54). DP thymocytes are a focal point in the process of T cell “selection and education” so that damaging or deleting them through interaction with infected TAPCs would almost certainly affect the emerging T cell repertoire and its ability to recognize foreign epitopes including those of viral and other pathogens. Figure 5C shows that thymocyte depletion in PRRSV-infected piglets does indeed produces “holes” in the T cell repertoire. Thus, a deficiency of peripheral helper T cells due to loss of precursors CD4^+^CD8^+^ thymocytes, even if random, could explain the delay in the appearance of high affinity antibodies that can neutralize PRRSV. Interestingly there are no reports of thymic atrophy or lymph node adenopathy in EAV and this arterivirus infection is typically resolved without persistence in contrast to PRRS (55). There are no reports of thymic atrophy in PCV-2 and SIV and as shown in Figure 1, these infection in isolator piglets are not associated with hypergammaglobulinemia.

In studies using isolator piglets, the level of CD4^+^CD8^−^ α/β cells in blood gradually decreases in piglets infected with the mildly pathogenic PRRSV VR-2332 compared to littermates infected with SIV and PCV-2 (34). This decrease is also seen in PRRSV-infected conventionally-reared piglets (28, 29, 43) especially using highly pathogenic strains (HP-PRRSV). Infection with both mild and HP-PRRSV strains also results in a decrease in CD8α^+^ γ/δ T cells. In both PRRS isolator piglets and conventional piglets, there is a sharp rise in CD4^+^CD8^+^ α/β T cells after 21–28 dpi. These peripheral DP cells, that express CD8α, have been regarded as either “activated” or “memory” helper T cells. The kinetics of the reciprocal decrease in CD4^+^ and increase in these DP α/β T cells might suggest this is merely a phenotypic/differentiation event in the same CD4^+^ T cells which may reflects a change in function. The increase in DP helper T cells parallels the increase in CD2^+^CD21^−^ AFC in the BAL suggesting they may drive terminal differentiation of B cells at this site, resulting primarily in IgA^+^ cells (34).

Discussion of thymic atrophy and thymocyte deletion also needs to consider the concept of central immune tolerance. During the Critical Window of Immunological Development (13), the offspring's immune system makes numerous decisions regarding how to respond to environmental and self-antigens and these depend heavily on the action of T cells. Therefore, any impairment of T cell development can impact both antibody and CTL development and function. There are several scenarios. First, PRRSV-infected TAPCs mediate random apoptosis of developing DP thymocytes including those that recognize all foreign epitopes including those of PRRSV and other pathogens. A second possibility is that the infected TAPCs present PRRSV epitopes as self-peptides to DP thymocytes triggering their apoptosis and thus eliminating them from the developing T cell repertoire. The mechanisms of DP elimination is the same as used in central tolerance induction. Such thymocytes deletion would be selective and dependent on the affinity of their TCR for particular PRRSV epitopes. A third possibility is that some elements of both mechanisms operate simultaneously to impair the ability of developing piglets to recognize PRRSV and other pathogens.

Both models can explain the persistence of PRRSV infections, the low levels of IL-4 and the CD4+ lymphopenia. The random depletion model is consistent with the increased susceptibility of piglets to other pathogens (14–17), and can explain why there are sufficient non-specific T cells to aid polyclonal B cell activation (9, 34). However, it cannot easily explain why pan-specific and anti-nucleocapsid (NC) responses are normal (23, 56) while VN antibody responses are consistently delayed (57). The last pattern is a better fit for the second scenario in that thymocytes with TCRs that tightly bind VN epitopes are deleted but not those to NC epitopes and others which bind poorly. It is well known that thymocytes with low affinity TCRs survive “thymic education.” In normal individuals, these comprise the autoreactive T cell subset. In our hypothesis and scenario, they are the DP thymocytes that bind poorly to NP T cell epitopes. The third scenario is a “fail-safe” argument which includes the central tolerance scenario, and can explain all available data.

In contrast to piglets, PRRSV induced depletion of DP thymocytes in adult swine would not prevent the production of the effective VN antibodies that can transfer protection (11, 12). Adult animals, not infected with PRRSV, fetally or neonatally, would have experienced normal, undisturbed thymus development and therefore, should have developed a normal peripheral T cell repertoire. This includes T cells able to help in the selection and proliferation of B cells with high affinity BCRs for VN epitopes. If thymocyte loss occurs in adult animals, it would be too late to seriously impair the T cell repertoire. Thus, there would be sufficient peripheral T cells to recognize PRRSV epitopes including those needed for the production of high affinity VN antibodies and those of other pathogens.

Since our hypothesis revolves around thymic atrophy, we need to mention that thymic atrophy occurs in many infectious diseases and often results in apoptosis of DP thymocytes. These typically comprise 80% of all thymocytes in mice and are under heavy selective pressure in the healthy thymus so that apoptotic cells are a normal feature of thymocyte development even in normal individuals. However, thymic atrophy and apoptosis is increased in AIDS, rabies, hepatitis, pestiviruses in cattle and swine and especially parasitic diseases (58–62). Infection of the mouse thymus by highly virulent influenza can also cause thymic atrophy (63). Similar to PRRS, thymic atrophy is positively correlated with virulence but interestingly in Chagas disease, a non-virulent strain of the parasite does not induce thymic atrophy (64). In most cases, the thymic epithelium is infected or damaged and elements of the extra-cellular matrix, e.g., laminin, collagen, etc. are deposited in higher than normal amounts. In parasitic infections and AIDS, macrophages and DCs are infected, similar to what is described for the TAPCs in PRRS. It appears that a number of infectious agents target the thymus, presumably to knock-out or dampen the host's specific immune recognition system and thereby allow the pathogen to thrive. Different pathogens may use somewhat different mechanisms to attack different elements of T cell development, but all with the same overall objective.

If thymic atrophy and interference with T cell development is common to so many infectious agents, why have PRRS researchers not examined this issue given that much older studies in mice showed that *in utero* infection with LCMV and hepatitis B lead to loss of virus-specific T cells (65, 66)? Since >25 years of PRRS research has failed to identify the immune pathology of the PRRS pandemic, we chose to remind investigators of the thymic atrophy which occurs in PRRS and to emphasize its effect on development of the T cell repertoire.

Much like thymic atrophy, polyclonal B cell activation and hypergammaglobulinemia are features of many viral infections including choriomeningitis virus (LCMV), LDV and HIV-1 (41, 67, 68). As with PRRS, only a small proportion of the excessive amount of IgG is specific for the virus (9). While this has not been measured for IgM and IgA, the lack of variable region diversity in PRRS (34, 36, 37) predicts it would affect all isotypes. The phenomenon seems to be the work of non-specific T cells including those promoting isotype switch (67) and has been observed for other viruses (68–70). While perhaps driven by LCMV, HIV-1, trypanosomes and PRRSV, class-switch recombination in swine occurs even during mid-gestation in the absence of environmental antigen or infection (71). In both PRRSV and HIV-1, infection results in a peripheral T cell deficiency. Either directly or indirectly, hypergammaglobulinemia and its ensuing events rely on some form of T cell, since TCRβ^−/−^ athymic mice and those lacking CD40L, do not develop hypergammaglobulinemia.

Numerous polyclonal B cell activators have been described including parasite proteins, Staphylococcal protein A, gp120 of HIV, envelope glycoproteins of LDV and various PAMPs acting through TLRs (41). While no candidate has been described for PRRS, unpublished reports that killed PRRSV can also activate B cells, and the virus presents a B cell superantigen, may supports the latter. Naturally cytokines are involved and since PRRSV infects macrophages, IL-6 is an obvious candidate. In IL-6 deficient mice infected with murine cytomegalovirus (MCMV), polyclonal B cell activation is reduced (72). In trypanosome-induced polyclonal B cell activation, stimulation of CD11b+ cells results in production of IL-6, IL-10, and BAFF (73). The consensus view in infectious diseases is that B cell differentiation is dependent on IL-6 and IL-1 derived from macrophages and dendritic cells. IL-15 may also be involved (74).

The polyclonal B cell activation that we observed in PRRSV-infected isolator piglets has another feature similar to what is seen with HIV-1. In HIV-1, GC formation is delayed or absent in gut-associated lymphoid tissues [GALT; (67)] so that B cell development proceeds without antigen selection in the mucosal immune compartment. Unfortunately, the current literature provides no quantitative or qualitative information about lymph node GC in PRRSV-infected piglets or adult swine, the latter which develop sterilizing immunity. Of interest is that LCMV which causes thymic atrophy and polyclonal B cell activation, is also associated with a delay in appearance of VN antibodies from 70 to 200 days (26) reminiscent of events in PRRS (24, 25).

Since polyclonal B cell activation is a common feature of various viral diseases and was pronounced in isolator piglets infected with PRRSV (Figure 1), it is worthwhile to ask whether it provides some protection to the host, favors the infectious virus or is just a distractive by-stander event. This subject was treated by Montes et al. (68) and the phenomenon summarized below. On the negative side, polyclonal B cell activation can be a distractive mechanism triggered by pathogens to lower the probability for pathogens to encounter a pathogen-specific B cell. On the positive side, it causes the production of natural antibodies that have a broad range of specificities, although their affinity for any particular pathogen might be low (37). Either scenario requires the host to expend energy to produce antibodies that are poorly designed to neutralize or eliminate the pathogen. Since polyclonal B cell activation appears to parallel a deficiency of antigen-specific T cells, it suggests that the phenomenon reflects a kind of “desperation” by the host, or “all-out-war” in hopes that the pathogenic threat can be handled using the “brute force” of natural antibodies.

A “dark side” feature of polyclonal activation is the appearance of autoantibodies. When secreted in large amounts, these can have pathological consequences. During “normal” immunological development of lymphocytes in fetal and neonatal vertebrates, autoreactive T cells are selected against during thymocyte development, an event which leads to *central tolerance*. One characteristic of arteriviruses is the production of autoantibodies to NP and the Golgi apparatus (9, 40). Establishing central tolerance depends on thymic TAPCs which in PRRS, are virus-infected. Hence, their ability to present antigen to the developing DP thymocytes may be impaired, allowing self-reactive T cells to leave the thymus along with those that consider PRRSV as “self.” Self-reactive B cells are de-selected in bone marrow during B cell lymphogenesis but unlike T cells, can be somatically generated throughout life. Nevertheless, their expansion and differentiation still depends on antigen-specific T cells. A low level of autoreactive T and B cells is normal and only foster autoimmune disease when their numbers are abnormally elevated by high concentrations of self-antigens, which may explain their detection in PRRS.

**Figure 6:**
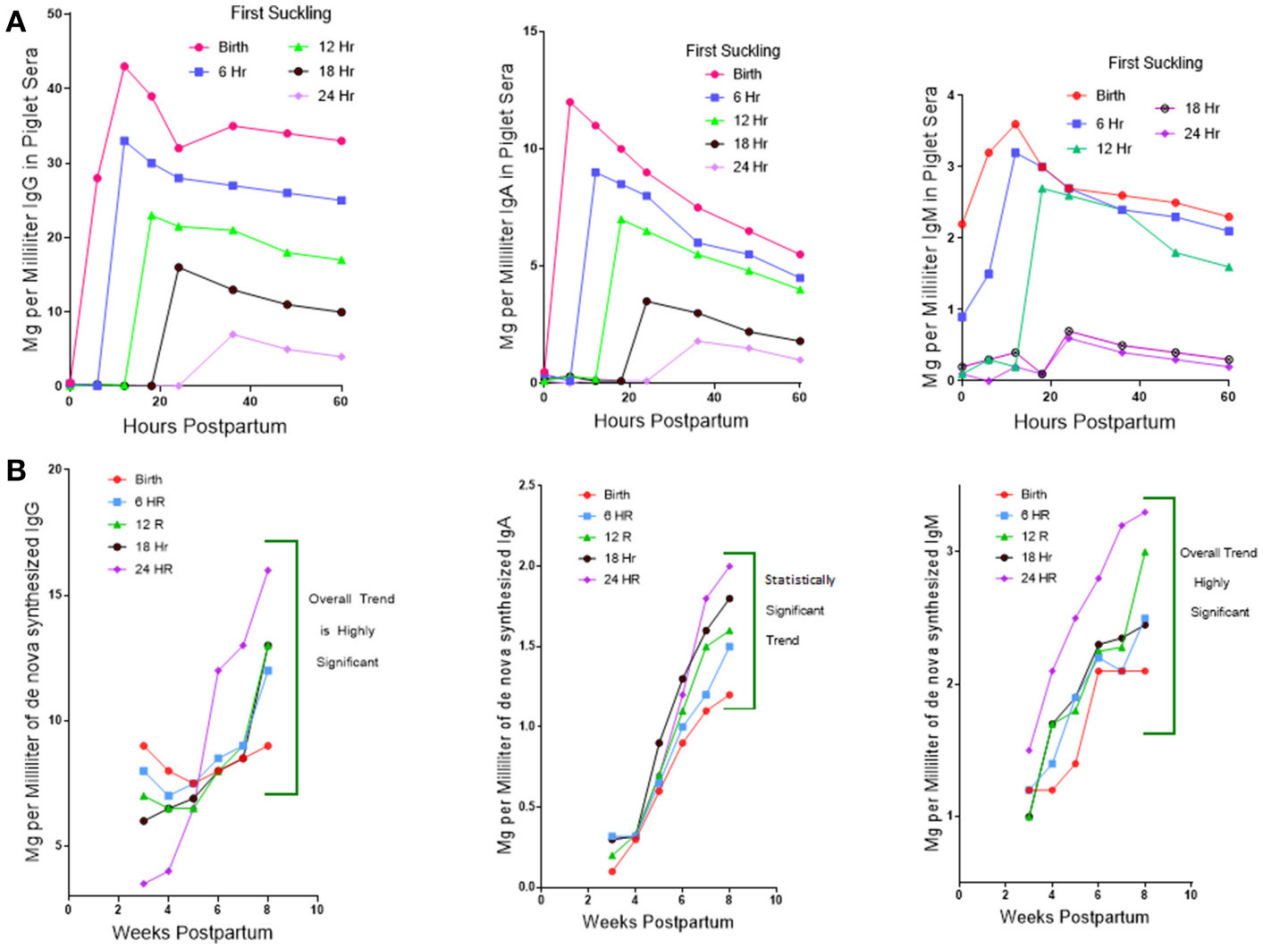
Regulation of *de novo* Ig synthesis in suckling piglets by IgG of maternal origin. **(A)** (Top). Serum IgG levels in piglets for which suckling was delayed in 6 h interval for up to 24 h. postpartum (PP). Legend on figure. Error bars eliminated to remove clutter. **(B)** (Bottom). The level of *de novo* synthesis of IgG, IgM, and IgA 4–8 weeks PP in piglets in which suckling was delayed at 6 h intervals for up to 24 h. Error bars removed to avoid clutter.

While investigators studying PRRSV infections in conventional piglets regularly report polyclonal B cell activation and lymph node adenopathy (75), none report the hypergammaglobulinemia seen in isolator piglets (Figure 1). Rather, limited and unpublished data suggest that IgG levels in PRRS after 4 weeks are similar to the norm reported for healthy piglets of the same age (Figure 1; see double arrows). The paucity of data on this point may be because investigators decided that hypergammaglobulinemia would be masked in conventionally reared piglets that ingested a bolus of maternal colostrum. This would be true if measurements were made before 25 days after birth but would no longer be true 4 weeks postpartum (PP) when >95% of serum Ig in piglets is of *de novo* origin (Figure 6). The alternative and more plausible explanation is that hypergammaglobulinemia is weak, absent or easily overlooked in PRRSV-infected conventionally-reared piglets. Unlike isolator piglets, they receive colostrum. Therefore, it is less interesting to speculate on what causes polyclonal B cell activation in PRRSV-infected isolator piglets and in other infections, than to address the question as to why polyclonal B cell activation does not progress to hypergammaglobulinemia in PRRSV-infected conventionally-reared piglets. This question is the basis of our second hypothesis.

**Figure 7:**
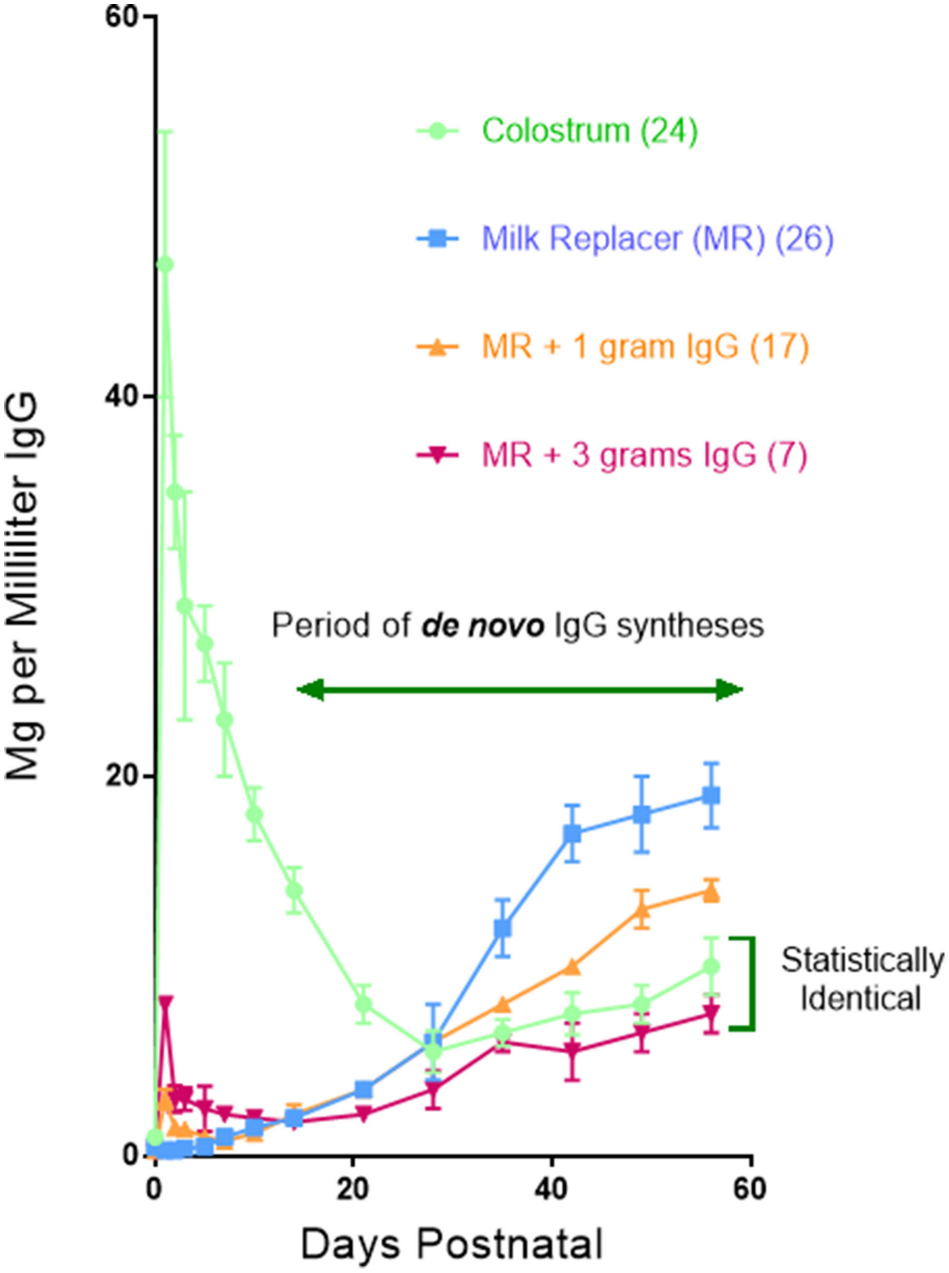
The concentration of IgG in the sera of piglets reared in an autosow facility. Piglets were reared on porcine colostrum or a bovine-based milk replacer (MR) to which each piglet received either 1 or 3 g of purified swine IgG during the first day. Legend is on the figure. Numbers in parentheses indicate the number of piglets in each group. Error bars are SEM. From Klobasa et al. (76).

A clue as to why hypergammaglobulinemia has not been reported in conventionally-reared piglets comes from work done 30–40 year ago (76). In these older studies, delaying suckling at 6 h intervals during the first 24 h. Postpartum (PP) resulted in a progressive decrease in the amount of maternal Igs that enter the blood of newborn specific pathogen free (SPF) piglets (Figure 6A). This occurs because gut closure starts at birth and progressively increases so by 24 h. PP only fragments of maternal IgG can be found in serum (77). Over the same period, the concentration of Igs in porcine colostrum progressively decreases from >90 mg/ml at birth to < 20 mg/ml at 24 h (78). In SPF piglets given a bovine-based milk replacer (MR) *de novo* Ig synthesis is spontaneous, apparently due to non-pathogenic environmental stimuli. At day 20 PP, 50% of serum IgG is of *de novo* origin, and this rises to 81% on day 25 PP and >95% on day 30 PP. Hence, at 4 weeks PP, virtually all serum Igs are of *de novo* origin. Figure 6B shows that *de novo* synthesis of IgG, IgM, and IgA after 4 weeks PP in suckling SPF piglets is inversely proportional to the amount of maternal IgG absorbed from colostrum (76). Since other factors in colostrum might explain this results, SPF piglets were reared in an autosow facility and raised on maternal colostrum or a bovine-based milk replacer (MR) to which purified swine IgG was added in various amounts. Figure 7 shows that the level of serum IgG after 8 weeks in piglets that received only the MR were two-fold higher that animals receiving colostrum and that administration of just 3 g total of purified swine IgG, reduced the level of *de novo* synthesis to that seen in piglets receiving colostrum [(76); Figure 6A vs. **B**]. Three grams is only 20% of the amount of IgG that is provided in colostrum, yet it is enough to suppress *de novo* synthesis to the level seen in piglets receiving porcine colostrum. The spontaneous increase in *de novo* IgG synthesis in piglets receiving only the MR was not associated with any clinical disease. IgG dependent down-regulation of B cell activity is well-documented (79, 80) and has been explained as idiotypic network regulation (81–84). The T15 idiotypic system has been described for those wishing to test the idiotypic regulation hypothesis in swine (85). It is noteworthy that piglets reared on the MR were ingesting and absorbing large amounts of all bovine Igs (86), yet this bovine IgG did not suppress *de novo* Ig synthesis in these piglets, an observation consistent with the view that few idiotypes cross species lines.

**Figure 8:**
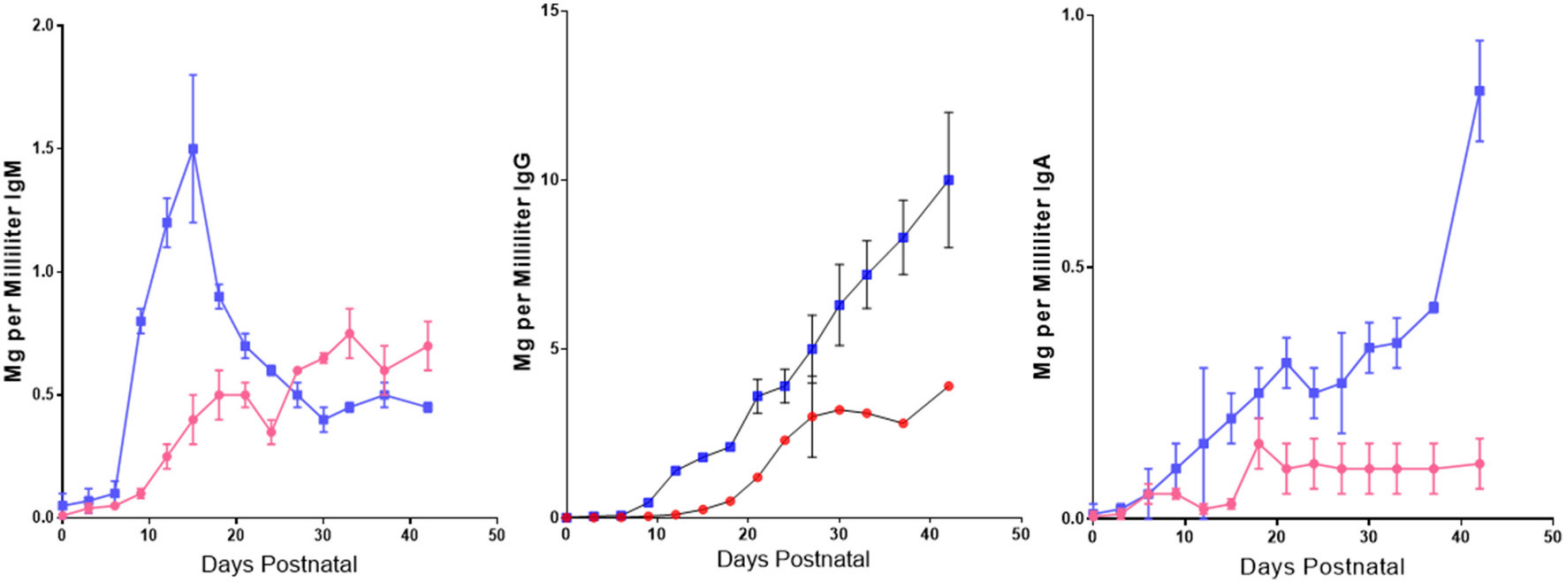
Effect of labile factors in colostrum on *de novo* Ig synthesis in piglets. Piglets were reared in an autosow facility on fresh colostrum or colostrum frozen −20 C for 1–8 years. Length of storage had no effect so data from all three storage times were pooled. 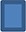 = stored (*n* = 24) 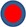 = fresh (*n* = 9). Error bars are SEM. Differences after 12 days PP for IgG and IgA were highly significant (*p* < 0.05) For IgM, differences were significant from 9 to 23 days PP (*p* < 0.05). From Klobasa et al. (86).

In addition to the effect of maternal IgG on *de novo* IgG synthesis in suckling piglets, it was also observed that colostrum stored at −20 C for 1 month to 8 years, prior to use in rearing SPF piglets in the autosow facility, resulted in substantial loss of down-regulatory capacity [(86); Figure 8]. Immunoglobulin levels were unaffected by storage over any time period. While storage has no significant effect 6–7 weeks PP on *de novo* IgM synthesis, it suppressive effect 2 weeks PP was striking (Figure 8). Loss of down-regulation at the time was ascribed to labile unidentified regulatory factors.

It was not until the period from the 1990s, that reports began to surface on the relatively high levels of cytokines in lacteal secretions of a number of species (33). Those in highest concentrations were TGFβ, IL-10, CSF, and EGF. All are major modulators of T cell activity and are delivered in large amounts to the newborn where they mediate the transformation of helper T cells into regulatory T cells (Tregs). Once referred to as “suppressor T cells,” these Tregs secrete both IL-10 and TGFβ that control B cell activation and expansion. These and other cytokines in porcine colostrum are absorbed into the sera of suckling piglets (33). These investigators also showed that TGFβ significantly reduced the ability of LPS to generate Ig secreting cells *in vitro*. In human infants these can dampen the host response to beneficial members of the gut microbiome and to food antigens like maternal milk proteins for which little central tolerance is developed. In the veterinary world, cows forced to “hold their milk” develop local edema which then forces milk proteins to enter the blood where they are recognized as foreign and can produce lethal anaphylactic shock (87). All four of the immune regulatory factors mentioned above are directly or indirectly immunosuppressive. Porcine, bovine and human colostrum deliver 40–400 micrograms of TGFβ daily to the newborn gastrointestinal tract (GIT). Porcine colostrum contains 1.5 mg/ml of EGF and when delivered experimentally, promotes the development of Tregs and also drives IgA switch recombination in the mucosal immune system (88). These regulatory factors have been shown to be important in pediatrics medicine and have been used in arguments supporting the importance of breast-feeding (89, 90).

In summary we believe the suppressive effect of colostrum (Figure 6) can be attributed to IgG (Figure 7) as well as to labile colostral factors (Figure 8) including cytokines like TGFβ and IL-10. These colostral factors can collectively explain why hypergammaglobulinemia has not been reported in conventionally-reared PRRSV-infected piglets.

This hypothesis article has mostly focused on the immunological pathway that normally results in VN antibodies which in turn leads to the establishment of sterilizing immunity. Our emphasis on the antibody aspect is because the delay in forming VN antibodies is a widely-cited feature by investigators and because almost every study on PRRS involves some measurement of antibody activity. However, any PRRSV-induced deficiency in the T cell repertoire would also cause a deficiency of the virus-specific CTLs that are needed to eliminate virus-infected cells. While less discussed, a CTL deficiency would equally contribute to persistence of the infection.

The purpose of this article has been to assimilate observations made in the past 25 years in an effort to develop a testable hypothesis that could move PRRS research forward and to encourage investigators to test it. Currently, investigators appear stuck on the very first step of the scientific method, i.e., the accumulation of phenomena such as thymic atrophy. We believed that construction of a testable hypothesis could help others to characterize the mechanism of PRRS-induced immune dysregulation and pathogenesis. We believe that the continued development of subunit vaccines or those based on attenuated virus, will remain a partial fix until the mechanism of immune dysregulation is known. Thus, we encourage PRRS investigators to design and perform experiments like those described in the next section of this article that can address critical tenets of our hypothesis. Should our hypothesis be confirmed, namely that the PRRSV interferes with development of the T cell repertoire in a manner that prevents the piglet from recognizing neonatal pathogens, it could shift emphasis to development of vaccines or other treatments that block this interference. Given that older animals develop sterilizing immunity, “holding the block” on the CD163 receptor on TAPCs or disrupting it until the piglet can complete development of their normal T cell repertoire, would be a step in the right direction.

## Materials and Methods

### Previously Published Data

With exception of some new data presented in Figure 5, all of the methods employed and materials used in obtaining the data presented are provided in the cited publications. In regard to Figure 5, samples collected during previously published experiment (34) from animals infected by PRRSV strain VR-2332 were used as a starting material. Frozen material included cDNA prepared using random hexamer primers on total RNA from cell suspensions isolated using TRI Reagent according to a protocol recommended by the manufacturer (Sigma-Aldrich). Analysis of these samples are described below.

### RNA Isolation, PCR Amplification and CDR3 Length Analysis

Each cDNA preparation was amplified in three concurrent analyses for one of three Vβ super-families (VβI–VβIII families, VβIV–VβVI families, and VβVII family) (34, 47). The 1st round PCR targeted the original cDNA preparation while the 2nd round PCR targeted the 1st round PCR products. After checking PCR product on 1.5% agarose gels stained by GelRed, the diversity of the Vβ CDR3 regions was inspected by CDR3 length analyses. Technically, the second round of PCR products for each Vβ superfamily was subjected to the 3rd round of PCR that involved incorporation of radioactive adenosine 5'-ATP γ-32P triphosphate nucleotide labeled Cβ primer (34). The products were separated on sequencing gels that were subsequently dried and their radioactive images were obtained by Storage Phosphor Screens BAS-IP MS scanned in fluorescent Image Analyser FLA-7000 (Fujifilm Corporation, Yokyo, Japan). All primers and PCR conditions used for amplifications were published earlier (34).

## Confirming Observations And Testing Hypotheses

### Verification of Basic Observations

Figures 4, 5 summarize key observations that underlie our major hypothesis. Before proceeding to undertake major and perhaps costly experiments to test it, we encourage others to confirm and extend our observations. Those with existing tissue specimens might compare the number of GCs in PRRSV-infected vs. a variety of control piglets. Such additional observations may help to justify more defined studies like those described below. Verification studies should also focus on establishing a more definitive phenotype for the cells we have referred to as TAPCs and which are positive for the PRRSV NC.

### Testing the Hypotheses

Described below are four studies that test tenets of our major hypothesis plus one that tests our secondary hypothesis.

1. PRRSV infection reduces the size of the specific T cell repertoire.

Construct a series of tetramer assays using T cell epitopes for PRRSV, SIV, and ovalbumin (OVA). The latter can be adapted from murine experiments. Infect groups of conventional and/or isolator piglets with moderately virulent PRRSV or SIV. Two to 3 weeks later, immunize half of each group plus uninfected controls with OVA in Freund's adjuvant. After boosting, determine for each group/subgroup the proportion of T cells recovered from various sites that recognize PRRSV, SIV, or OVA in tetramer assays. We predict that a lower proportion of T cells recognize PRRSV than SIV and that PRRSV infection, but not infection with SIV, significantly reduces the number of OVA-specific T cells.

2. Loss of T cells in PRRSV-infected piglets is selective.

Should it be shown in the above experiment that thymic atrophy is associated with a reduced functional T cell repertoire, it is important to determine whether this has been selective for PRRSV epitopes. Using the same tetramer methods or something equivalent, the T cell repertoire of infected piglets and control piglets should be compared using a spectrum of candidate T cell epitopes such as those for NC and GP5 and others considered important for providing help in production of VN antibodies. We predict that deletion of T cells from the repertoire will be selective and may favor deletion of those involved in the production of VN antibodies.

3. Helper T cell-rich germinal centers (GCs) are greatly reduced in PRRS.

Newborn SPF piglets should be infected with PRRSV or SIV, and sacrificed 20 dpi or at 30 dpi if challenged with virus or vaccine. Immunohistochemical analyses of peripheral lymphoid tissues, especially including the tracheal bronchial lymph nodes, can them be examined for germinal centers using AID as a marker and appropriate mAbs to test for co-localization of DP, activated T cells.

4. Non-neutralizing antibodies in PRRS are of low affinity.

Using several purified PRRSV B cell epitopes especially recognized VN epitopes, develop a BiaCore or modified ELISA assay to measure absolute and/or relative affinity of IgG antibodies produced by conventional PRRSV-infected piglets. Develop similar assays for SIV. We predict that the antibodies to VN epitopes of PRRSV will be of lower affinity than those to SIV and when used in *in vitro* VN assays, they will behave poorly compared to those against SIV.

5. Colostral factors reduce hypergammaglobulinemia in PRRSV-infected piglets.

This experiment is designed to test our secondary hypothesis that colostral factors down-regulate B cell activity in newborn piglets. Recover two or more litters of colostrum-deprived, Caesarian derived (CDCD) piglets from genetically diverse backgrounds and rear them under SPF conditions. Allow a mixture of half to suckle a surrogate sow. Infect half of the piglets in the suckling group and half in the non-suckling group with a mildly pathogenic PRRSV. Measure serum IgG levels on a daily/weekly schedule. We predict that serum IgG levels 30 days postpartum in PRRSV-infected piglets allowed to suckle will be significantly lower than in PRRSV-infected piglets denied colostrum. This experiments allows for numerous permutation and advanced studies that are not discussed here.

## Author Contributions

GW, YL, and XC contributed and organized Figures 4, 5 (in part). JB drafted the manuscript and contributed Figures 1, 3, 6, 7, 8. MS and KS provided the data summarized in Figures 2, 5 and its interpretation.

### Conflict of Interest Statement

The authors declare that the research was conducted in the absence of any commercial or financial relationships that could be construed as a potential conflict of interest.
